# Systemic Ozone Therapy Improves Oral Hard and Soft Tissue Healing in Medication-Related Osteonecrosis of the Jaw (MRONJ): A Study in Senescent Female Rats

**DOI:** 10.3390/biomedicines13051248

**Published:** 2025-05-20

**Authors:** Leonardo Alan Delanora, Tiburtino José de Lima Neto, Tiago Esgalha da Rocha, Glauco Rodrigues Carmo Silveira, Liran Levin, Jamil Awad Shibli, Edilson Ervolino, Carlos Fernando Mourão, Leonardo P. Faverani

**Affiliations:** 1 Department of Diagnosis and Surgery, School of Dentistry, São Paulo State University (UNESP), Araçatuba 16015-050, Brazil; leonardo.delanora@unesp.br (L.A.D.); faverani@unicamp.br (L.P.F.); 2 Department of Oral Diagnosis, Piracicaba Dental School, University of Campinas (UNICAMP), Piracicaba 13414-903, Brazil; limaj@unicamp.br; 3 Department of Basic Sciences, School of Dentistry, São Paulo State University (UNESP), Araçatuba 16066-840, Brazil; tiago.esgalha@unesp.br (T.E.d.R.); glauco.silveira@unesp.br (G.R.C.S.); e.ervolino@unesp.br (E.E.); 4 Faculty of Dentistry, University of Saskatchewan, Saskatoon, SK S7N 5E5, Canada; liran.levin@usask.ca; 5 Dental Research Division, Department of Periodontology and Oral Implantology, University of Guarulhos (UNG), Guarulhos 07023-070, Brazil; jshibli@ung.br; 6 Department of Basic and Clinical Translational Sciences, School of Dentistry, Tufts University, Boston, MA 02111, USA

**Keywords:** osteonecrosis, ozone, tooth socket, wound healing, bisphosphonate-associated osteonecrosis of the jaw

## Abstract

**Background/Objectives**: Medication-related osteonecrosis of the jaw (MRONJ) is a challenging condition often associated with bisphosphonate use, leading to impaired bone healing and difficult clinical management. Given the lack of predictable therapeutic options, this study investigated the effects of systemic ozone therapy on MRONJ healing. This study aimed to analyze the effects of systemic ozone therapy on oral hard and soft tissue healing in senescent rats with medication-related osteonecrosis of the jaw (MRONJ) induced by antiresorptive therapy. **Methods**: Twenty-eight senescent Wistar rats, aged eighteen months and weighing ~350 g, were used for this study. The animals were divided into four groups. The negative control (SAL) group received saline applications, while the control-treated (SAL+OZ) group received saline applications and ozone therapy (0.7 mg/kg). The MRONJ (ZOL) group received Zoledronate, an intravenous antiresorptive drug (100 μg/kg), and the MRONJ-treated (ZOL+OZ) group received zoledronate application and was treated with systemic ozone therapy (0.7 mg/kg). All rats underwent molar extraction in the third week of the experiment and were euthanized in the seventh week of the experiment. The mandibles were resected, reduced, and prepared for microtomographic analysis, histopathological/histometric analysis, and immunohistochemistry. **Results**: The ZOL group presented characteristics of vitreous, non-vital, and dense bone, poor vascularization, and high values of inflammation markers compatible with MRONJ. In contrast, the ZOL+OZ group exhibited improvement in alveolar bone and soft tissue healing, a decrease in nonvital bone area, and modulation of local inflammation. **Conclusions**: It can be concluded that Ozone therapy improved oral hard and soft tissue healing of MRONJ in senescent female rats subjected to antiresorptive drugs and might be considered for future clinical applications.

## 1. Introduction

Antiresorptive drugs are widely used to treat osteopenic conditions, bone metastases, multiple myeloma, Paget’s disease, and other bone-related disorders [1]. Bisphosphonates (BPs), in particular, play an important therapeutic role in controlling and preventing these conditions [1]. However, they have been related to an adverse effect on bone necrosis of the jaw, referred to as medication-related osteonecrosis of the jaw (MRONJ) [2].

MRONJ affects approximately 1% to 12% of patients receiving intravenous antiresorptive therapy [2,3], with incidence increasing to 21% after three years of continued use due to cumulative tissue deposition [3]. These drugs are primarily prescribed for elderly patients and postmenopausal women with osteoporosis, which contributes to the rising prevalence of MRONJ in these populations [1,2]. The most common site of occurrence was detected in the posterior part of the mandible, between the first molar and first premolar [3]. Tooth extraction has been identified as a major local risk factor for MRONJ development [1,2].

Since the initial reports of MRONJ [4], numerous studies have been published, including systematic reviews and preclinical investigations [2,5]. However, its etiopathogenesis remains unclear, limiting the establishment of a predictable, gold-standard treatment protocol [1,3].

Ozone has shown promising results in terms of tissue healing optimization. It presents as a gas under standard temperature and pressure conditions, has a cyclic structure, and comprises three oxygen atoms. It has been used in different ways, such as in gas insufflation and oily constitutions or dissolved in solutions [6]. It has been shown to improve tissue response and blood circulation. Furthermore, it enhances the transport of blood oxygen, modulates antioxidant enzyme activity, and regulates the function of the immune cells. It stimulates angiogenesis, the production of growth factors and fibroblasts [7,8].

MRONJ not only impairs quality of life but also compromises oncologic and osteometabolic treatments due to its potential complications and resistance to conventional management strategies [1,3,4]. Current therapies often yield unpredictable outcomes and do not adequately restore tissue function [5]. Therefore, identifying adjunctive or alternative approaches that can modulate inflammation, promote vascularization, and support bone and soft tissue healing is of high clinical relevance. Ozone therapy, due to its reported regenerative and immunomodulatory effects, emerges as a promising candidate to address these challenges [6].

Given the limited evidence supporting effective treatments for MRONJ [1,2,5], this study aimed to investigate the effects of systemic ozone therapy in a senescent rat model of MRONJ.

## 2. Materials and Methods

### 2.1. Study Design and Ethical Considerations

This research followed all the precepts and ethical principles for animal experiments (ARRIVE 2.0) [9]. All experimental protocols were approved by the Ethics Committee on the Use of Animals (CEUA) of the School of Dentistry of Araçatuba—São Paulo State University/UNESP (# 00498-2019) on 25 June 2019.

Animals were randomly assigned to the experimental groups using a simple randomization method. All outcome evaluations (histological, immunohistochemical, and imaging) were performed by blinded, calibrated examiners to minimize observer bias.

Twenty-eight senescent rats (*Rattus novergicus*, Wistar), aged eighteen months and weighing ~350 g, were included. These rats were evenly distributed into four groups (n = 7): Group 1 (SAL), the negative control group, received 0.45 mL of 0.9% sodium chloride solution (saline) intraperitoneally every three days for seven weeks. In group 2 (ZOL), osteonecrosis was induced with the application of 100 µg/kg zoledronate every three days for seven weeks (positive control). Group 3 (SAL+OZ) received intraperitoneal saline every three days for seven weeks plus intraperitoneal ozone therapy at a dose of 0.7 mg/kg every two days for four weeks (starting the day of the tooth extraction). In group 4 (ZOL+OZ), osteonecrosis was induced with zoledronate in the same protocol mentioned above, with ozone therapy following the same application model above every two days for four weeks (starting the day of tooth extraction). The doses and drug treatment plans used were adapted for rats but are similar to those used in cancer therapy in humans [10,11].

Throughout the experiment, the animals were kept in the vivarium of the Department of Diagnosis and Surgery in plastic cages suitable for the species in an environment with a stable temperature (22 ± 2 °C) and a controlled light cycle and were fed a solid feed and water ad libitum.

The intervention started on day one, with the installation of a cotton thread ligature around the animals’ first molar to imitate the extraction of a tooth due to periodontal disease [10,12,13,14].

After extraction, the rats belonging to the SAL+OZ and ZOL+OZ groups received ozone therapy intraperitoneally every 2 days, starting on postoperative (PO) day 0 until day 28. The ozone was administered using the device provided by the company OZONE & LIFE INDÚSTRIA, COMÉRCIO E SISTEMAS LTDA (São José dos Campos—SP, Brazil), which was also used to establish the dosage of 0.7 mg/kg.

All procedures that could cause some discomfort or pain in the animals were performed under general anesthesia using ketamine (70 mg/kg, Francotar, Virbac, Hortolândia, SP, Brazil) and xylazine (10 mg/kg, Rompum Bayer, Coxilha RS, Brazil) by intramuscular injection [10,11]. Animals were monitored daily for general health and behavioral changes throughout the experiment. Efficient surgical techniques and humane handling were employed to minimize stress and discomfort.

Three weeks after the start of medication, the rats were anesthetized, the cotton ligature was removed, and oral antisepsis was performed with PVPI 10% (Riodeine Degermante, Rioquímica, São José do Rio Preto, Brazil). The first lower left molar was extracted [10,11].

All rats were sacrificed 28 days after the extraction of teeth. The rats were anesthetized as mentioned above and submitted to cardiac perfusion with 100 mL of saline added with 0.1% heparin and 800 mL of 4% formaldehyde in phosphate-buffered saline (0.1 M, 4 °C, pH 7.4) [10].

All surgical procedures were performed under general anesthesia to prevent pain, and animals were monitored daily for general health and behavioral changes throughout the experiment. Efficient surgical techniques and humane handling were employed to minimize stress and discomfort.

### 2.2. Computed Microtomography (MicroCT)

Animal jaws were removed from the fixative and washed for 12 h in water and then were scanned using a digital computerized microtomography system. The parts were digitalized using the SkyScan device (SkyScan 1176 Bruker MicroCT, Aatselaar, Belgium, 2003) with 9 µm thick cuts (90 Kv and 111 μA). The images obtained were stored and reconstituted to determine the area of interest using the NRecon software (SkyScan, 2011; Nrecon Version-1.6.6.0 Software/Bruker Corporation, Kontich, Belgium) [15].

Using the DataViewer software (SkyScan, Version 1.4.4 64-bit—Skyscan Dataviewer/Bruker Corporation, Kontich, Belgium), the images were reconstructed to standardize positioning for all samples in three planes (transverse, longitudinal, and sagittal). Then, using the CTAnalyzer software (CTAn—SkyScan, 2012 Bruker MicroCT Version 1.12.4.0, Karlsruhe, Germany), the radiographic area of interest (ARI) was defined, which was delimited by an area of 4 mm^3^ between the root apex of the left first molar and the portion of bone superior to the root of the central incisor. The CTAn software (SkyScan, 2012 Bruker MicroCT Version 1.12.4.0, Karlsruhe, Germany) was used to analyze and measure the images in grayscale. The threshold set was 40–160 shades of gray [15].

### 2.3. Analysis and Processing of the Soft Tissues

After demineralization using 10% ethylenediaminetetraacetic acid (EDTA) for eight weeks, the histological sections of the portion of the dental socket and adjacent tissues to be analyzed were collected from the lingual and vestibular regions. After microtomy, the pair slides were stained with hematoxylin and eosin (HE) [10,11].

### 2.4. Microscopic Analysis and Microscopic Area of Interest (AMI)

Microscopic analyses were performed by a certified histologist (E.E) who was blinded to the treatment groups following the same method used for the analyses carried out in previous studies [10,11].

### 2.5. Histopathological and Histometric Analysis

Histological analysis of the tooth extraction site and adjacent areas was performed by evaluating the following parameters: (1) intensity of the local inflammatory response, (2) extension of the inflammatory process, (3) cellular pattern and epithelium tissue structure, (4) cellular pattern and connective tissue structure, and (5) cellular pattern and bone tissue structure. Each of these parameters was categorized according to the histological cellular aspects shown in Table 1 [11].

Histometric analysis was performed to determine the percentage of newly formed bone tissue (PONF). The percentage of non-vital bone tissue (PONV) was determined through photomicrographs of the area of the extracted tooth alveolus and adjacent tissues using image analysis software (Axiovision 4.8.2 Carl Zeiss—Carl Zeiss™ AxioVision Rel. 4.8.2 Software for Use With: Light microscopes, PC Digital Film and Video Imaging and Documentation Components, Fisher Scientific—Göttingen, Germany) [10,11].

### 2.6. Immunohistochemical Analysis

The immunohistochemical processing and analysis followed the protocol previously described by Ervolino et al. [11] using the immunoperoxidase technique. The odd histological sections were subjected to indirect immunoperoxidase using the following primary antibodies: anti-TNF-α (SC-1348, Santa Cruz Biotechnology—Orlando, FL, USA), anti-IL-1β (SC-1252, Santa Cruz Biotechnology—Orlando, FL, USA), anti-VEGF (SC-7269, Santa Cruz Biotechnology—Orlando, FL, USA), anti-OCN (SC-18319, Santa Cruz Biotechnology—Orlando, FL, USA), and anti-TRAP (SC-30833, Santa Cruz Biotechnology—Orlando, FL, USA) [10,14].

Photomicrographs were collected from the connective tissue of the mucosa overlying the dental extraction site in the immunohistology sections. Using the same image analysis program (Axiovision 4.8.2), measurements were made of the area occupied by bone tissue at the dental extraction site. These values were expressed per mm^2^ [11].

### 2.7. Statistical Analysis

All data were subjected to the Shapiro–Wilk test for normality. The parametric values passed the analysis of variance (Two-way ANOVA, and One-way ANOVA) tests, and when they showed a significant difference, multiple comparisons were performed using the Student–Newman–Keuls post-test. For the non-parametric parameters, the Kruskal–Wallis test was used, and the Student–Newman–Keuls post-test was used when *p* < 0.05. The statistical program used was SigmaPlot version 12.0 (Exakt Graphs and Data Analysis, San José, CA, USA), considering *p* < 0.05. All analyses were conducted with consideration of data distribution, and the choice of test for each dataset was based on the outcome of normality and variance assessments.

## 3. Results

### 3.1. Analysis of the Volumetric Parameters (MicroCT)

**Figure 1:**
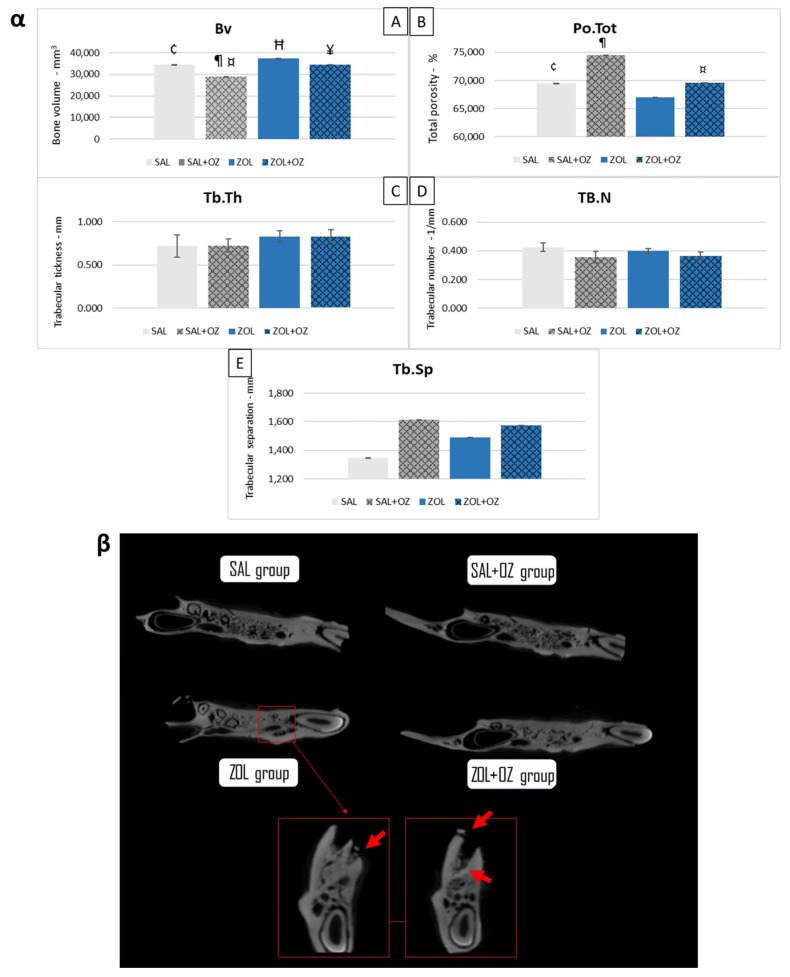
(**α**) Graphical representation of the means and standard deviations of the volumetric parameters: (Bv)—(**A**), (Po.Tot)—(**B**), (Tb.Th)—(**C**), (Tb.N)—(**D**), and (Tb.Sp)—(**E**) of the experimental groups SAL, SAL+OZ, ZOL, and ZOL+OZ. BV = bone volume, Tb.Th = bone trabecular thickness, Tb.Sp = separation of bone trabeculae, Tb.N = number of trabeculae, and Po.tot = percentage of total porosity. Where “¶” represents the statistical difference between ZOL and SAL+OZ, “Ħ” represents the statistical difference between ZOL and SAL, “¥” represents the statistical difference between ZOL and ZOL+OZ, “¤” represents the statistical difference between ZOL+OZ and SAL +OZ and “¢” represents the statistical difference between SAL and SAL+OZ. (**β**) Comparative axial tomography section of experimental groups, where it is possible to observe the density difference, specifically a higher hyper density in the ZOL group. Red arrows show a bone sequestrum resulting from the deficient healing process and the non-remodeling of the extraction site—MRONJ.

The ZOL group presented a higher bone volume than the SAL group (*p* < 0.05). The SAL+OZ group had the lowest values and presented less bone volume than all the other groups (*p* < 0.05). The ZOL+OZ group had a lower mean than the ZOL group (*p* < 0.05). However, the ZOL+OZ group obtained similar values to the SAL group (Figure 1α).

In terms of total porosity (Po.Tot), the ZOL group had the lowest average value compared to other groups. The ZOL+OZ group showed higher bone porosity than the ZOL group, but this was not a statistically significant increase (*p* = 0.07).

The values of bone trabecular thickness (Tb.Th), number of trabeculae (Tb.N), and separation of bone trabeculae (Tb.Sp) were similar in all groups. However, the data showed trends indicating denser bone for the groups treated with zoledronate (ZOL and ZOL+OZ). The bone density can be seen in the microtomographic comparisons in Figure 1β in addition to bone sequestrations from MRONJ disease in the ZOL group.

### 3.2. Histopathological and Histological Analysis

**Figure 2:**
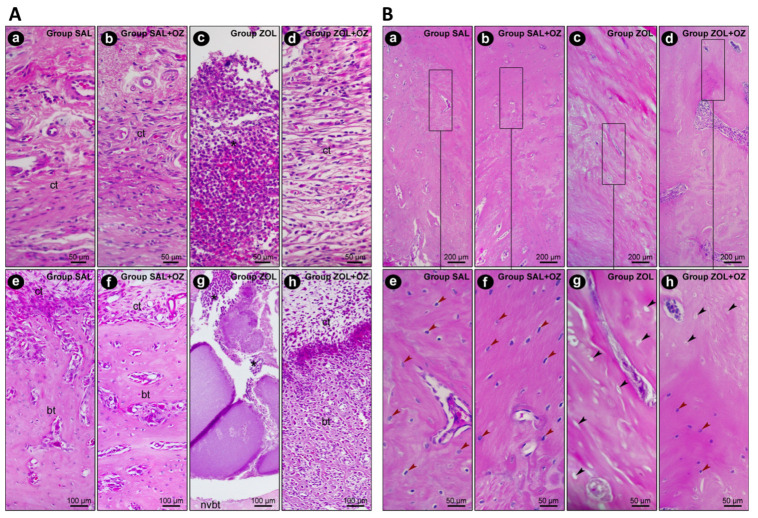
(**A**) Representative photomicrographs of the suprabony/supracrestal soft tissue (**a**–**d**) and the interior of the dental extraction site (**e**–**h**) at 28 postoperative days of the experimental groups SAL (**a**,**e**), SAL+OZ (**b**,**f**), ZOL (**c**,**g**), and ZOL+OZ (**d**,**h**). Abbreviations and symbols: (*) inflammatory infiltrate; ct: connective tissue; bt: bone tissue; nvbt: non-vital bone tissue. Staining: HE. Original magnification: 400× (**a**–**d**) and 200× (**e**–**h**). Scale bars: 50 and 100 μm. (**B**) Representative photomicrographs of the adjacent bone of the dental extraction site at 28 postoperative days of the experimental groups SAL (**a**,**e**), SAL+OZ (**b**,**f**), ZOL (**c**,**g**), and ZOL+OZ (**d**,**h**). Abbreviations and symbols: Red arrows show osteoblastic lineage cells; black arrows show empty osteocyte gaps, with cellular absence. Staining: HE. Original magnification: 100× (**a**–**d**) and 400× (**e**–**h**). Scale bars: 200 and 50 μm.

In terms of the histopathological analysis of the first molar extraction site and adjacent tissues, the ZOL group presented the highest values regarding inflammation intensity and extension and tissue damage (*p* < 0.05), as can be seen in Figure 2. The ZOL+OZ group showed improvement in all parameters compared to the ZOL (*p* < 0.05); Table 1.

The presence of vital bone tissue in the SAL and SAL+OZ groups, followed by the ZOL+OZ group, was evident in the high-quality histological sections. Even with some gaps in the bone matrix, the number of cells of the osteoblastic lineage was noticeable, with osteocytes embedded in the mineralized bone matrix and osteoblasts in the adjacent region of the bone tissue (bt), juxtaposed to the connective tissue (ct) loaded with cells. The difference between the ZOL group and the other groups was evident, where several empty gaps were visible, which is a typical characteristic of non-vital bone tissue (nvbt); (Figure 2).

**Figure 3:**
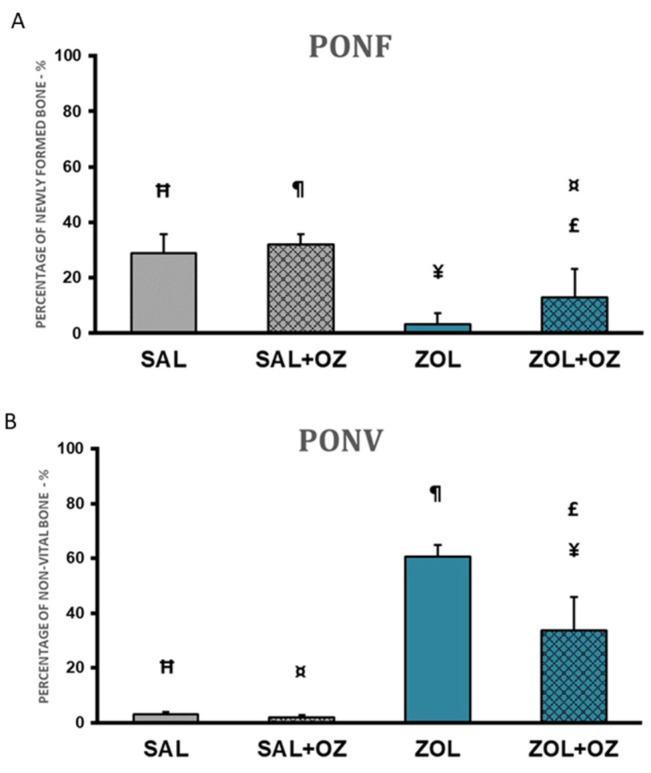
(**A**) Graphical representation of the means and standard deviations of the percentage of newly formed bone comparing the experimental groups SAL, SAL+OZ, ZOL, and ZOL+OZ. (**B**) Graphical representation of the means and standard deviations of the percentage of non-vital bone in the experimental groups SAL, SAL+OZ, ZOL, and ZOL+OZ. Abbreviations and symbols: PONF: Percentage of newly formed bone. PONV: Percentage of non-vital bone. “¶” represents the statistical difference between ZOL and SAL+OZ, “Ħ” represents the statistical difference between ZOL and SAL, “¥” represents the statistical difference between ZOL and ZOL+OZ, “¤” represents the statistical difference between ZOL+OZ and SAL +OZ, “£” represents the statistical difference between ZOL+OZ and SAL.

The highest percentages of newly formed bone in the extraction site were found in the SAL+OZ group, followed by the SAL group, ZOL+OZ group, and ZOL group. The ZOL group showed a statistically significant difference from all the other groups (*p* < 0.01). The mean of the ZOL+OZ group also differed significantly from SAL (*p* < 0.05) and SAL+OZ (*p* = 0.01); (Figure 3A).

A large amount of non-vital bone tissue was observed in the ZOL group compared to the SAL and SAL+OZ groups (*p* < 0.01). The amount of non-vital bone found in the ZOL+OZ group was significantly different from the ZOL, SAL, and SAL+OZ groups (*p* < 0.05; Figure 3B).

### 3.3. Immunohistochemistry

**Figure 4:**
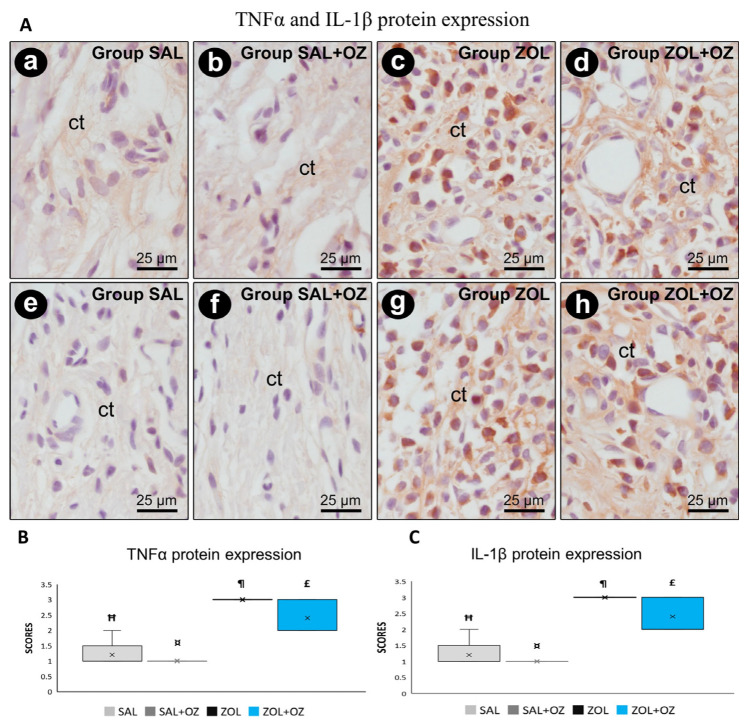
(**A**) Photomicrographs showing the pattern of immunostaining for TNFα at the dental extraction site at 28 postoperative days in SAL (**a**), SAL+OZ (**b**), ZOL (**c**), and ZOL+OZ (**d**) as well as for IL-1β in SAL (**e**), SAL+OZ (**f**), ZOL (**g**), and ZOL+ OZ (**h**). Abbreviations and symbols: ct connective tissue. Counterstain: hematoxylin. Original magnification: 1000×. Scale bars: 25 µm. (**B**,**C**) Graphical representation of protein expression: TNFα (**B**) and IL-1β (**C**) in experimental groups SAL, SAL+OZ, ZOL, and ZOL+OZ. Where “¶” represents the statistical difference between ZOL and SAL+OZ, “Ħ” represents the statistical difference between ZOL and SAL, “¤” represents the statistical difference between ZOL+OZ and SAL +OZ, and “£” represents the statistical difference between ZOL+OZ and SAL.

Higher scores of TNFα and IL-1β were observed in the ZOL and ZOL+OZ groups, where both showed a statistically significant difference from the SAL and SAL+OZ groups (*p* < 0.05). Representative photomicrographs as well as the median and statistical differences are shown in Figure 4.

**Figure 5:**
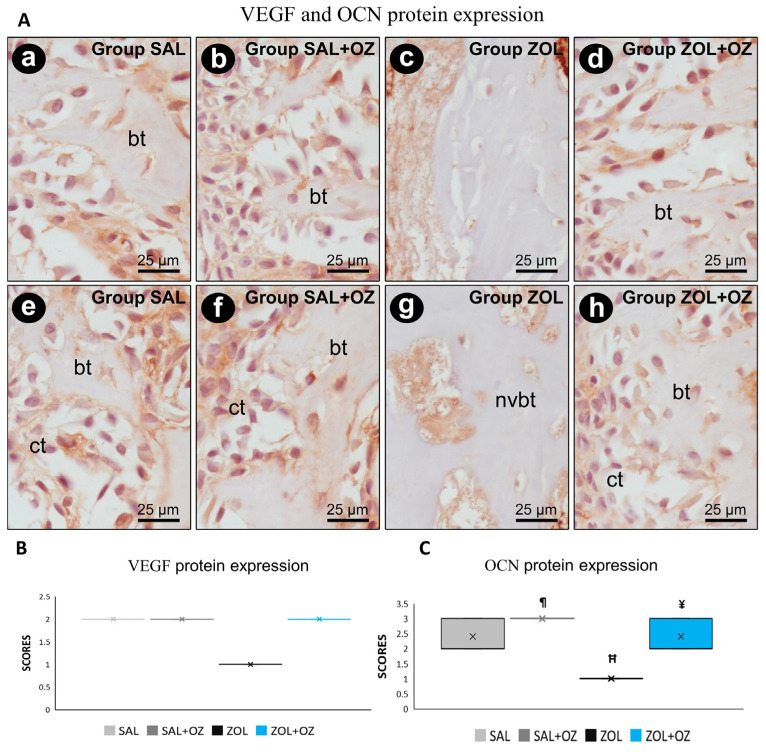
(**A**) Photomicrographs showing the pattern of immunostaining for VEGF at the dental extraction site at 28 postoperative days in SAL (**a**), SAL+OZ (**b**), ZOL (**c**), and ZOL+OZ (**d**) as well as for OCN in SAL (**e**), SAL+OZ (**f**), ZOL (**g**), and ZOL+OZ (**h**). Abbreviations and symbols: ct: connective tissue; bt: bone tissue, nvbt: non-vital bone tissue. Counterstain: hematoxylin. Original magnification: 1000×. Scale bars: 25 µm. (**B**,**C**) Graphical representation of protein expression: VEGF (**B**) and OCN (**C**) in experimental groups SAL, SAL+OZ, ZOL, and ZOL+OZ. Where “¶” represents the statistical difference between ZOL and SAL+OZ, “H” represents the statistical difference between ZOL and SAL, and “¥” represents the statistical difference between ZOL and ZOL+OZ.

For VEGF and OCN labeling, the lowest scores were observed for the ZOL group, visible in the slides with low immunostaining of endothelial cells and osteoblastic cells (Figure 5). The photomicrographs representative of the expressions as well as the median and statistical differences are shown in Figure 5.

**Figure 6:**
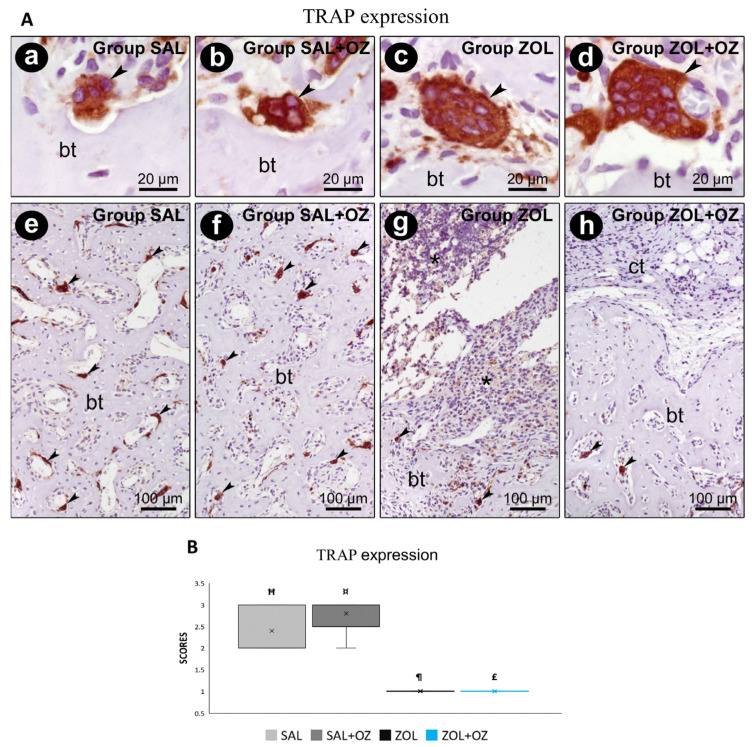
(**A**) Immunostaining for TRAP at the dental extraction site and its surroundings on the 28th postoperative day. Photomicrographs showing the immunostaining pattern for TRAP-positives (osteoclasts) at the dental extraction site at 28 postoperative days in SAL (**a**,**e**), SAL+OZ (**b**,**f**), ZOL(**c**,**g**), and ZOL+OZ (**d**,**h**). Abbreviations and symbols: (*) inflammatory infiltrate; ct: connective tissue; bt, bone tissue; arrows: osteoclast. Counterstain: hematoxylin. Original magnification: 2000× (**a**–**d**) and 200× (**e**–**h**). Scale bars: 20 µm (**a**–**d**); 100 µm (**e**–**h**). (**B**) Graphical representation of TRAP staining in experimental groups SAL, SAL+OZ, ZOL, and ZOL+OZ. Where “¶” represents the statistical difference between ZOL and SAL+OZ, “Ħ” represents the statistical difference between ZOL and SAL, “¤” represents the statistical difference between ZOL+OZ and SAL +OZ, and “£” represents the statistical difference between ZOL+OZ and SAL.

For TRAP labeling, a greater number of marked osteoclasts were observed in the control groups (SAL and SAL+OZ). The lowest scores were obtained for groups treated with BPs (ZOL and ZOL+OZ). The results of these groups were significantly different from the groups without zoledronate administration (SAL and SAL+OZ; *p* < 0.05; Figure 6).

## 4. Discussion

There is still no consensus on a safe and efficacious treatment for patients who are affected by MRONJ [1,2,7,16]. When comparing the animals in the experimental group that underwent zoledronate treatment for seven uninterrupted weeks (ZOL group) and the control group that received only a saline solution for seven weeks, changes in the bone architecture due to bisphosphonate were evident. This validates the hypothesis that bisphosphonate, together with bone physiology, takes a certain amount of time to affect the bone architecture [17].

MicroCT analysis revealed statistically significant differences between the ZOL and ZOL+OZ groups. This demonstrates the effectiveness or at least the modulatory potential of ozone therapy on bone architecture, where ozone was found to act systemically by braking or modulating this bone “petrification” characteristic of zoledronate [6,9,18]. In healthy animals (SAL+OZ), systemic ozone therapy led to reduced bone volume and increased porosity, likely reflecting a transient phase of active bone remodeling rather than pathological loss. This behavior may be associated with ozone-induced stimulation of physiological turnover in aged bone tissue.

It is important to note that the high values of bone parameters found in the microtomography of the ZOL group should not be read as high values of healthy bone or vital bone [18]. Although bone volume increased in the ZOL group, the reduced porosity (*p* = 0.07) and histological features support the interpretation of pathological densification rather than functional bone regeneration.

This was evident from the histopathological and histometric analyses, in which the ZOL group showed a higher amount of non-vital bone tissue, a characteristic of MRONJ [2,3,7]. Due to the faster metabolism in rats compared to humans, this occurred in two to three weeks compared to more than eight weeks as observed in humans [11,19]. Soft tissue healing was confirmed by improved epithelial continuity, reduced inflammatory infiltration, and more organized connective tissue in the ZOL+OZ group when compared with the ZOL group, supported by histological scores and reduced IL-1β and TNF-α expression.

Histopathological and immunohistochemical analyses showed very high expression of proinflammatory proteins TNFα and IL-1β in groups treated with BPs (ZOL and ZOL+OZ). This finding aligns with the higher amount of non-vital bone tissue observed in the ZOL group, since inflammatory cells are attracted to non-vital tissue and lead to exacerbated tissue damage [20], which, in association with a cavity rich in pathogens, increases the occurrence of MRONJ [1,3,7,10].

Bone turnover is directly related to the activity and homeostasis between osteoblasts and osteoclasts. However, exposure to BPs drastically affects bone turnover, as can be seen in the analysis of osteocalcin (OCN) and TRAP in the ZOL and ZOL+OZ experimental groups [20,21]. This can be interpreted as a deficiency in bone deposition and mineralization by osteoblasts, which, when active, are the main secretors of OCN. The groups that were administered zoledronate showed the lowest expression of TRAP, which can be attributed to the reduced osteoclast activity, which can affect the physiological bone remodeling process so that static bone eventually deteriorates and can result in tissue death [20,21].

Moreover, the ZOL+OZ group showed a significant difference compared to the ZOL group in terms of OCN protein expression. It did not present a statistically significant difference compared to the SAL group. It appears that ozone therapy might be effective in the activation of osteoblasts and their functions. This can be directly linked to the values found in the PONF and PONV, where even with the expressive action of zoledronate, ozone therapy was able to reduce the amount of non-vital bone tissue and increase the presence of newly formed bone [6,16] The enhanced bone formation and reduced non-vital bone observed in the ZOL+OZ group suggest that ozone therapy mitigates ZOL-induced damage by reducing inflammation, restoring vascularization, and promoting osteoblast function.

The presence of non-vital and avascular bone in the ZOL group was supported by reduced VEGF immunolabeling [22]. The SAL and SAL+OZ groups had the highest markings for VEGF, followed by ZOL+OZ. This indicated the absence of vascularization in the ZOL group, a crucial factor for the nutrition of cells [2,6,22]. Interestingly, a statistical difference was found between the ZOL+OZ and ZOL groups, where ozone therapy increased VEGF protein expression, translated into neo-vasculogenesis, increased blood supply to the bone tissue, decreased the almost pathological bone density caused by zoledronate, and attracted new cells and precursors to the region, which could undergo differentiation and become osteoblasts. Therefore, ozone-induced VEGF expression likely facilitated bone regeneration through enhanced angiogenesis and recruitment of osteogenic precursors to the injury site. These findings correlate with the expression of OCN [16,22].

The animals subjected to therapy with ozone (SAL+OZ and ZOL+OZ) exhibited greater new bone formation than their untreated counterparts. This is probably due to the characteristics of cell bioactivation, which stimulate cell differentiation and the production of energy for these cells, among other pathophysiological responses [10,11]. In addition, the antioxidant properties of ozone help keep cells younger and healthier compared to those without treatment, not to mention the neoangiogenic capacity, which stimulates the formation of new vessels that will eventually transport both constitutive cells and essential nutrients and oxygen to the tissue [6,7,8,23]. In addition, improved epithelial thickness, connective tissue organization, and decreased expression of TNF-α and IL-1β in the ZOL+OZ group provide consistent evidence of enhanced soft tissue healing as promoted by systemic ozone therapy.

Compared to other adjunctive approaches, such as platelet-rich plasma, low-level laser therapy, or antimicrobial protocols, systemic ozone therapy offers distinct advantages [2,24]. These include low procedural morbidity, improved tissue perfusion, and the ability to be applied at a distance from the lesion site [25,26]. Such characteristics are particularly relevant for patients with limited mouth opening, postoperative anatomic restrictions, or tumor-related mutilations, where access to intraoral lesions is challenging [3,5].

One of the primary objectives of research involving animal models is to generate translational data that can be extrapolated to clinical practice. In this study, the selection of female rats reflects the higher incidence of the disease reported in various studies, with proportions of up to 75% affecting the female sex [1,2,3]. In addition, the age of senescent animals (18 months old) corresponds to the age of most patients affected by MRONJ, who are typically over 55 years old in most reports [2,3,10]. In the literature, there are very few reports of the disease affecting young adults under 24 years of age, which may be attributed to rapid metabolism and significant local angiogenesis due to systemic compensation [19,24]. Studies conducted with young male rats (3–6 months old) as animal models may yield therapy outcomes that are not translatable to clinical practice [25]. This is because these rats exhibit rapid tissue repair behavior and minimal metabolic changes, even when subjected to high doses of antiresorptive drugs [27]. It is still necessary to use experimental models that analyze more critical conditions, as observed in most patients with MRONJ, so that the results can be more accurate and reliable. However, we would like to stress the point that translational research can provide better information, such as the model shown in this present study, which is the most clinically relevant model that we can provide.

No adverse effects were noticed in the animals that used the ozone therapy, but specific studies for this purpose should be conducted. These findings are in agreement with several previous studies [6,7,8,23,26].

In contrast to most existing studies, which focus on topical or localized ozone application, our study provides evidence of the systemic effects of ozone in a senescent animal model with MRONJ-like features. These findings highlight ozone’s ability to act beyond local modulation, impacting both bone and soft tissue repair through systemic immunomodulatory and pro-angiogenic mechanisms. This is particularly relevant considering that MRONJ predominantly affects elderly or medically compromised patients, in whom systemic therapeutic approaches are often necessary. This expands the understanding of ozone’s therapeutic range and supports its investigation as a systemic adjunct in osteonecrosis management.

There is a need for further studies, especially controlled trials aiming to provide a reliable basis for the use of ozone as therapy for MRONJ [16,17,18,19]. It is important to emphasize that the present study has some limitations, such as the use of an experimental animal model that does not fully replicate the complexity of human MRONJ, including systemic comorbidities or chronic drug exposure. To overcome these limitations, future studies should consider using larger animal models, extended observation periods, and molecular analyses to better understand the long-term effects and mechanisms of systemic ozone therapy. Nonetheless, the findings from this model offer promising translational value and may inform the design of future clinical protocols targeting patients with impaired healing capacity. Nevertheless, the current findings provide a solid foundation for continued investigation, including different modes of application, so that ozone therapy may eventually be integrated into clinical protocols when appropriate.

## 5. Conclusions

The use of systemic ozone therapy suggested a positive modulation in the area of newly formed bone, with decreased non-vital bone tissue, improved local vascularization, modulated inflammatory responses of the tissues, and improved bone volumetric parameters for the groups using the treatment protocol. These effects, especially in a compromised healing model, suggest that systemic ozone may have clinical potential as an adjunctive strategy to enhance bone and soft tissue repair in MRONJ. These findings provide a rationale for future studies and may support the development of novel therapeutic strategies.

## Figures and Tables

**Table 1:** Parameters, scores, and distribution of specimens according to histological analysis of tissue after tooth extraction in different experimental groups. Symbols: * a: statistically significant difference in relation to SAL; * b: statistically significant difference in relation to SAL+OZ; * c: statistically significant difference in relation to ZOL.

Histological Analysis				
Parameters and Respective Scores	Percentage of Specimens			
	Experimental Groups			
	SAL	SAL+OZ	ZOL	ZOL+OZ
**Intensity of local inflammatory response**				
**(1)** absence of inflammation (presence of few inflammatory cells)	100%	100%	-	60%
**(2)** mild quantity of inflammatory cells (less than 1/3 of cells are inflammatory cells)	-	-	-	40%
**(3)** moderate quantity of inflammatory cells (1/3–2/3 of cells are inflammatory cells)	-	-	40%	-
**(4)** severe quantity of inflammatory cells (more than 1/3 of cells are inflammatory cells)	-	-	60%	-
**median**	**1**	**1**	4*a,b	1*c
**Inflammation extension**				
**(1)** absence of inflammation	100%	100%	-	60%
**(2)** partial extension of connective tissue	-	-	-	40%
**(3)** entire extension of connective tissue, without reaching bone tissue	-	-	20%	-
**(4)** entire extension of connective tissue and bone tissue	-	-	80%	-
**median**	**1**	**1**	4*a,b	1*c
**Cellular pattern and epithelial tissue structure**				
**(1)** epithelial tissue with moderate thickness (larger than half the surgical wound epithelium border thickness) and completely recovering extraction site	60%	80%	-	20%
**(2)** epithelial tissue with thin thickness (smaller than half the surgical wound epithelium border thickness) and completely recovering extraction site	40%	20%	-	80%
**(3)** thin layer of epithelial tissue (smaller than half the surgical wound epithelium border thickness) and only in edges of open surgical wound	-	-	20%	-
**(4)** absence of epithelial tissue on open surgical wound	-	-	80%	-
**median**	**1**	**1**	4*a,b	2*c
**Cellular pattern and connective tissue structure**				
**(1)** moderate quantity of fibroblasts and large quantity of collagen fibers (approximately 2/3 of area occupied by fibroblast/collagen fibers, where collagen fibers are prevalent over fibroblasts)	60%	80%	-	20%
**(2)** moderate quantity of both fibroblasts and collagen fibers (approximately 2/3 of area occupied by fibroblast/collagen fibers, where collagen fibers and fibroblasts are equivalent)	40%	20%	-	80%
**(3)** small quantity of both fibroblasts and collagen fibers (approximately 1/3 of area occupied by fibroblast/collagen fibers, where collagen fibers and fibroblasts are equivalent)	-	-	40%	-
**(4)** severe tissue disorganization with necrosis areas (approximately 2/3 of area occupied by disorganized connective tissue)	-	-	60%	-
**median**	**1**	**1**	4*a,b	2*c
**Cellular pattern and bone tissue structure**				
**(1)** absence of non-vital bone in adjacencies of extraction site and trabecular bone filling more than half of dental alveolus	80%	100%	-	-
**(2)** absence of non-vital bone in adjacencies of extraction site and trabecular bone filling less than half of dental alveolus	20%	-	-	60%
**(3)** presence of few areas with non-vital bone in adjacencies of extraction site and trabecular bone filling less than a third of dental alveolus	-	-	20%	40%
**(4)** presence of many areas with non-vital bone in adjacencies of extraction site and trabecular bone filling less than a third of dental alveolus	-	-	80%	-
**median**	**1**	**1**	4*a,b	2*a,b,c

## Data Availability

Data are available from the authors upon reasonable request and with the permission of L.A.D.
